# A profile of osteopathic practice in Australia 2010–2011: a cross sectional survey

**DOI:** 10.1186/1471-2474-14-227

**Published:** 2013-08-01

**Authors:** Sharyn R Burke, Ray Myers, Anthony L Zhang

**Affiliations:** 1 Discipline of Osteopathy, School of Health Sciences, RMIT University, Plenty Rd, Bundoora, Australia; 2 Discipline of Chinese Medicine, School of Health Sciences, RMIT University, Plenty Road, Bundoora, Australia

**Keywords:** Osteopathy, Osteopathic medicine, Cross-sectional survey, Scope of practice, Clinical presentations

## Abstract

**Background:**

There is limited evidence available to describe a profile of osteopathic practice in Australia. The purpose of this study was to describe the current activities of Australian osteopaths, using an internationally-developed standardised data collection tool.

**Methods:**

A voluntary national paper-based survey using a modified UK-developed standardised data collection tool was distributed to and completed by osteopaths across Australia between June 2010 and June 2011.

**Results:**

Fifty four osteopaths participated in this study and returned a total of 799 patient records. Two thirds of patients were female, with a median age of 39 years and age range of 7 days - 89 years. Almost three quarters of people seeking osteopathic care were employed and the largest source of referral was by word-of-mouth.

The majority of presenting complaints were acute musculoskeletal in nature. Approximately 38% of patients presented with a coexisting condition; the highest incidences were found in the cardiovascular and respiratory systems, along with mental health disorders.

Main treatment approaches were soft tissue (22.3%), muscle energy technique (14.6%), articulation techniques (14.3%) and education/advice (11.9%). Improvement or resolution of the complaint was experienced by 96.2% of patients within a small number of treatments. Complications of treatment were minor and of low frequency.

**Conclusions:**

In this study, Australian osteopaths mainly see patients with acute or sub-acute musculoskeletal problems which are predominantly spinal conditions. A significant proportion of these patients have one or more co-existing condition, largely of the cardiovascular and respiratory systems, along with mental health disorders. The majority of patients have a significant improvement within few treatments, with infrequent and minor adverse events reported. These findings should be tested through multi-centred pragmatic trials of osteopathic practice.

## Background

Osteopathy in Australia was the fastest growing complementary health profession at the last reported national census, with the reported number of osteopaths having tripled in number between 1996 and 2006 [1,2]. According to the Australian Bureau of Statistics, the number of people visiting osteopaths in the two weeks prior to the National Health Surveys of 1995 and 2005 increased by 88% to 60,000 [1]. Despite this, the scope of practice of the osteopathic profession appears to be unknown to many other health care practitioners, as well the general public, and it is not defined in any legislation in Australia.

Osteopathy originated in the USA in 1874, and the development of the profession there led to trained practitioners, known as ‘osteopathic physicians’, having full and unlimited practice rights, with their scope of practice equivalent to medical practitioners [3]. The scope of practice of the osteopathic physician in the USA is well understood; osteopathic physicians are regarded as “parallel and distinctive” in regulatory and legislative affairs, as well as being included in consultation for most issues of public health policy [4].

In countries such as Australia, Britain and New Zealand, practitioners have limited practice rights [3]. Osteopathy emerged from the USA in the early part of the twentieth century to these countries [3], however the individual legislations of these countries did not permit the full licence practice of osteopathy as undertaken in the USA, and this difference resulted in the appearance of two recognised groups – the osteopath, and the osteopathic physician. Practitioners with limited practice rights, such as those in Australia, Britain or New Zealand, are described as ‘osteopaths’ , as opposed to ‘osteopathic physicians’ [3]. Both groups share the same fundamental philosophies and principles of osteopathic medicine, and the same core competencies [3]; however their scopes of practice vary significantly. Despite this disparity, Carreiro and Fossum [3] suggest that both osteopaths and osteopathic physicians have achieved “nationally recognised academic and professional standards within their country” that allow them to provide diagnosis and treatment based on the principles of the osteopathic philosophy.

In Australia, osteopathy became a regulated health profession in 1979, first in Victoria, followed by the remaining states [5]. Since 1986, the formal academic training of osteopaths has been university level, government-funded qualifications. Prior to this, all osteopaths practicing in Australia received their osteopathic qualifications from private colleges either locally or overseas.

In July 2010, the Health Practitioner Regulation National Law Act 2009 (The Act) was established, bringing the ten Australian regulated health professions together under one registration agency known as the Australian Health Practitioner Regulation Authority (AHPRA) [6], with the formation of the Osteopathy Board of Australia (OBA), which replaced the seven state-based regulatory schemes for osteopathy. The Act, (Section 38, 2b) provides for endorsements for recognised and defined areas of practice, but does not require mandatory definition of scope of practice [7]. The scope of practice definition should be informed by, but not limited to, the activities in which the profession is currently engaged. At this time, there is limited published literature [8-11] available which profiles Australian osteopathic activities in practice, and given the recent growth trend of the profession, this evidence is likely to be out of date.

In 2004 the Australian Osteopathic Association (AOA) conducted a national postal snapshot survey of 341 members’ practice activities on a single day, with the aim to “identify and characterise the profession” [8,12]. The survey sought details on both practitioners, and patients (n = 2238), and included demographic information, qualifications, work patterns, clinical activities, and patient characteristics such as presenting complaints, diagnosis and treatment modalities [8,9]. In 2005 Xue et al. [10,11] conducted a study on the uses of Complementary and Alternative Medicine (CAM) by Australians, which included a specific section on osteopathy, covering the prevalence of the use of osteopathy, demographic characteristics of patients, presenting conditions, referral frequency, and perceived benefits and adverse effects of treatment.

These studies found that the majority of patients attending osteopathic consultations were female and mainly presented with spinal pain syndromes. The most common areas were low back or neck pain, other spinal pain and headaches [8,9]. The main techniques employed were soft tissue technique, articulation, high velocity manipulation, muscle energy technique (MET), and exercise prescription. Technique descriptions are consistent with the Glossary of Osteopathic Terms in *Foundations of Osteopathic Medicine*[13]. The majority of patients believed the treatment had been helpful, reporting that treatment had relieved pain and symptoms. Adverse events were reported to be both minor and rare [8,10].

In 2009, the National Council for Osteopathic Research in Britain (NCOR) developed and trialled a Standardised Data Collection tool (SDC tool) to gather patient-based data within osteopathic private practice across the UK [14]. The initiatives for this trial were to characterise practice, set standards for audit activities, provide information relevant to the profession at large and direct focused research [14]. The trial demonstrated that the standardised data collection tool generated a substantial amount of high-quality data, which met the trial objectives, and was therefore thought to perform well overall [14]. The tool itself gathered information on patient demographics, presenting symptoms, patient management and treatment, and outcomes.

The purpose of this study was to use the UK-developed SDC tool, modified for the Australian context, to survey osteopaths across Australia about patient characteristics and practice activities, including demographic information, presenting complaints, co-morbidities, use of treatment modalities, treatment outcomes and adverse events, so as to report on the current profile of osteopathic practice in Australia.

## Methods

Approval for this project was granted by the RMIT University’s Human Research Ethics Committee.

### Design

A voluntary, paper-based survey was distributed to osteopathic clinics across Australia between June 2010 and June 2011.

### Recruitment

An e-flyer advertising the study was forwarded by the Australian Osteopathic Association (AOA) to all current members. Approximately 90% of registered osteopaths in Australia are represented by the AOA [15] and so recruitment of participants from this source was considered representative of the national osteopathic population. To encourage involvement, the e-flyer was sent to all AOA members (approximately 1350 members) on 3 occasions at fortnightly intervals in the initial recruitment period, and on a further 6 occasions during the data collection period. The researchers also offered an analysis of the participant’s data to be returned to them, and the AOA offered continuing professional development (CPD) points to participants. Participants were identified to the researchers and to the AOA administrative team (for the purposes of CPD point allocation). Interested osteopaths contacted the researchers directly by email or telephone. Any osteopath who held current national registration and who was in clinical practice (consulting with patients) was eligible to participate. Informed consent was assumed by participation in the study.

### Survey tool

Permission to use the SDC tool was granted by NCOR. The UK SDC tool comprised of two distinct sections, a section on demographic information, and a section on patient presentations, treatment, management and outcomes. A small number of questions on the tool were modified to suit Australian conditions. Modified questions reflected differences in Australian practice and socio-demographic information, including ethnicity and healthcare resources. Patients could not be identified by the researchers from the data collected; each record was given a numerical code specific to the participant, for the purposes of individual participant data analysis.

The modified tool was made up of 55 items (Additional file 1). The participant completed all sections of the form. The first section covered demographic information about the patient, including age, gender, ethnicity, occupation, height, weight, disability status, and information about the presenting complaint. Other key questions included symptom areas, onset, the presence of any co-existing conditions, referral route, and duration of presenting complaint.

The second section covered items regarding the initial treatment and management of the patient, and included treatment approaches used, information regarding consent, education, advice and self-management strategies for the patient, and payment. Further to this, information about second and subsequent appointments was collected in this section, including outcomes of treatment and occurrence of adverse events after administration of treatment.

The final section of the SDC tool collected information regarding the final visit within the data collection period, including complications of treatment or adverse events, overall improvement, total number of treatments received, follow up and final management, where relevant.

### Data collection

Each participant was sent a package by post containing 20 copies of the SDC tool, information guidelines on how to complete the forms, and prepaid envelopes for returning the completed forms. In addition, an advertisement and a copy of the survey tool were included to be placed within the participant’s clinic reception area to alert patients that the clinic was participating in the project. A patient then had the option to decline to have their data included in the study.

Participants were asked to complete the SDC forms on patients attending their clinic as either a new patient, or an existing patient presenting with a new complaint. The data collection period for each participant was three months from the date they were sent the SDC package. Each participant was sent a reminder email for returning completed forms at the 1 month, 2 month and 3 month intervals in the data collection phase. At the end of the study period, any participant with outstanding surveys was sent a final email requesting return of all forms, either complete or incomplete.

### Information guidelines

Participants were asked to complete the forms by filling in the information regarding the demographics and presenting complaint at the first appointment. They were then asked to follow through with the patient’s treatment and management until the end of the consultation course, or until the end of the three month data collection period, whichever came first. At this point, the participant was asked to complete the final two sections of the form for each patient, and then return the forms in the prepaid envelopes.

Participants informed patients about the data collection at the first consultation and any patient who did not want their data collected could choose not to be involved. Participants could contact the researchers at any time during the data collection period if they had any queries regarding the study. At the end of the three-month data collection period, each participant was asked for comments on the use of the SDC tool. This feedback was collected to inform future development of the data collection process.

### Participants: practitioners

In a separate online survey through Survey Monkey, participants were asked to provide details of their qualifications, number of years in practice, fulltime or part time practice, relevant postgraduate education and location of practice.

### Statistical analysis

Descriptive statistical analysis (percentage), correlations and cross tabulations of data (chi square test) were conducted using SPSS 20.0.

## Results

### Participants: practitioner information

Of the 74 registered osteopaths who responded to the recruitment e-flyer and agreed to participate, 54 returned data for this study. Twenty osteopaths failed to submit any data for the following main reasons: 1) more work than they anticipated; 2) difficulty in completing the tool; 3) other factors not related to the survey, including personal and health reasons, which prevented participation after the recruitment stage.

The number of respondents was small, at 3.4% of the target population, however the distribution of participants by state in this study was not significantly different to the distribution of AHPRA-registered osteopaths in Australia for 2011(*χ*^2^ = 0.60 p = 0.74) [12,16] (Table 1). Thirty six practitioners responded to the online survey with information regarding their practice and qualifications. This information was not found to differ significantly from the AOA Census data gathered in 2004 [2,8,9,12] other than that the location of their osteopathic training in Australia increasing from 85% to 97.2% (*χ*^2^ = 9.79 p < 0.01) and their work status changed from 54% to 72% (*χ*^2^ = 10.86 p < 0.01) in full time employment.

**Table 1:** **Characteristics of practitioner participants in this study and AOA census data/AHPRA statistics **[12,16]

**Characteristic**	**Survey participant (Practitioner) n (%)**	**AOA census data/AHPRA n (%)**	***χ***^**2 **^**(P value)**
**Gender**			
Male	24 (44.4)	156 (46.2)	0.07 (0.80)
Female	30 (55.6)	182 (53.8)	
Region *			
Victoria	27 (50.0)	715 (44.8)	
New South Wales	16 (28.6)	514 (32.2)	0.60 (0.74)
Other states & Territories	11 (20.4)	366 (23.0)	
**Age**			
20-29 years	10 (27.8)	126 (37.1)	
30-39 years	16 (44.4)	105 (30.9)	
40-49 years	6 (16.6)	66 (19.4)	3.77 (0.44)
50-59 years	4 (11.2)	37 (10.9)	
> 60 years	0 (0.0)	6 (1.8)	
**Location of training Institute**			
Australia	35 (97.2)	290 (85.0)	4.22 (0.04)
International	1 (2.8)	51 (15.0)	
**Years in practice**			
5 years or less	12 (33.3)	141 (44.1)	
Between 6–10 years	11 (30.6)	61 (19.0)	
Between 11–15 years	9 (25.0)	44 (13.8)	9.34 (0.053)
Between 16–20 years	1 (2.8)	34 (10.7)	
Greater than 20 years	3 (8.3)	40 (12.4)	
**Nature of employment†**			
Full time	26 (72.0)	178 (53.8)	4.92 (0.03)
Part time	10 (28.0)	153 (46.2)	

*Comparison to AHPRA data in 2011.

†For the purposes of this table, people reporting work hours greater than 32 are considered to be Full time.

### Patient records

#### Patient demographic information

A total of 799 patient records were collected from the 54 osteopaths who participated. The distribution of the demographic data recorded in this study did not differ significantly from the AOA census data (Table 2) [8,9,12].

**Table 2:** Socio-demographic characteristics of patients attending for osteopathic treatment (N = 799) and AOA census data

Characteristic*	**Patient records n (%)**	**AOA census n (%)**
**Gender (n = 790)**		
Male	256 (32.4)	(37.6)
Female	534 (67.6)	(62.4)
**Age (years) (n = 771)**		
0-9	68 (8.8)	(3.8)
10-19	25 (3.2)	(4.7)
20-29	114 (14.8)	(12.1)
30-39	226 (29.3)	(23.2)
40-49	142 (18.4)	(23.2)
50-59	109 (14.1)	(18.5)
60-69	54 (7.0)	(8.1)
70-79	25 (3.2)	(4.7)
80+	8 (1.0)	(1.8)
**Region (n = 779)**		
New South Wales	234 (30.0)	(32.7)
Victoria	359 (46.1)	(44.1)
Other states & Territories	186 (23.9)	(23.2)
**Employment (n = 768)**		
Employed	533 (69.4)	(70.3)
Unemployed	71 (9.2)	(1.5)
Not in labour force	164 (21.4)	(28.2)
**Ethnic background (n = 799)**		
Australasia	633 (79.2)	(88.4)
Overseas	166 (20.8)	(11.6)
**Source of referral (n = 799)**		
Self/word-of-mouth	570 (71.3)	(70.5)
General Practitioner	58 (7.3)	(4.9)
Other Healthcare Practitioner	78 (9.8)	(10.1)
Other	93 (11.6)	(14.5)
**Previous osteopathic treatment**	339 (42.8)	
**Appointment waiting time (n = 784)**		
Same day	229 (29.2)	n/a
2-3 days	346 (44.1)	n/a
4-7 days	118 (15.1)	n/a
8 or more days	91 (11.6)	n/a

*Number and % excluding patient records where data was not included.

### Clinical presentations

The data collection form allowed for up to three symptom areas to be recorded. Of the 799 records, 68.2% reported 3 symptom areas. Of the total symptom areas reported, complaints in the cervical spine were most prevalent (16.0%; n = 326), followed by lumbar spine (15.6%; n = 316), pelvis (14.2%; n = 289), lower limb (13.9%; n = 282) and upper limb (13.4%; n = 273). Combined, spinal presentations made up 41.5% (n = 843) of presenting complaints (Table 3).

**Table 3:** **Clinical presentations for patients in this study and AOA 2004 census data **[12]

**Characteristic**	**Patient records n (%)**	**AOA census n (%)**	**Patient records n (%)**	**AOA census n (%)**	**Patient records n (%)**	**AOA census n (%)**	**Patient records n (%)**	**AOA census n (%)**
**Site of complaint**	**1º**		**2º**		**3º**		**Total**	
Abdominal	5 (0.6)	18 (0.9)	6 (0.9)	11 (0.8)	6 (1.1)	9 (1.8)	17 (0.8)	38 (1.0)
Cervical	147 (18.6)	514 (24.4)	110 (15.8)	252 (18.8)	69 (12.7)	86 (16.7)	326 (16.0)	852 (21.5)
Head/Face	96 (12.1)	205 (9.8)	55 (7.9)	79 (5.9)	36 (6.6)	32 (6.2)	187 (9.2)	316 (8.0)
Lower limb	101 (12.8)	228 (10.8)	101 (14.5)	205 (15.3)	80 (14.7)	99 (19.3)	282 (13.9)	532 (13.4)
Lumbar	183 (23.1)	574 (27.3)	71 (10.2)	226 (16.8)	62 (11.4)	108 (21.0)	316 (15.6)	908 (22.9)
Pelvis	76 (9.6)	108 (5.1)	118 (16.9)	89 (6.6)	95 (17.4)	26 (5.1)	289 (14.2)	223 (5.6)
Ribs/Chest	21 (2.7)	112 (5.3)	22 (3.2)	148 (11.0)	19 (3.5)	44 (8.5)	62 (3.0)	304 (7.7)
Thoracic	52 (6.6)	157 (7.5)	82 (11.7)	185 (13.8)	67 (12.3)	35 (6.8)	201 (9.9)	377 (9.5)
Upper limb	84 (10.6)	184 (8.7)	111 (15.9)	146 (10.9)	78 (14.3)	75(14.6)	273 (13.4)	405 (10.2)
Other	26 (3.3)	4 (0.2)	22 (3.2)	2 (0.1)	33 (6.1)	0 (0.0)	81(4.0)	6 (0.2)
Total	791 (100.0)	2104 (100.0)	698 (100.0)	1343 (100.0)	545 (100.0)	514 (100.0)	2034 (100.0)	3961 (100.0)

The chronicity of the presenting complaint in this study was reported and of the total complaints, 48.2% (n = 385) of patients reported complaints that had been present for less than 4 weeks, with 36.4% (n = 291) present for greater than 12 weeks. For 40.1% (n = 316) of patients presenting for osteopathic treatment in this study, this was the first time they had experienced their complaint.

Coexisting health conditions in this study were found to be present in 37.9% of patients (n = 303). Conditions most prevalently reported included those of the cardiovascular system (17.4%; n = 139), mental health disorders (10.5%; n = 84), respiratory system (8.6%; n = 69) and arthritic conditions (7.1%; n = 57). Approximately one in nine patients (11.0%; n = 88) presented with more than one coexisting health condition.

### Treatment approaches

Of the 799 patients whose information was recorded in this study, 4 were deemed unsuitable for treatment at the first consultation, and a further 15 patients did not receive treatment at the first consultation, despite being considered suitable for treatment. The data collection tool did not seek an explanation as to why the patient was either not suitable for, or did not receive treatment.

Participants were asked to select treatment modalities used during consultation; they had the option of selecting as many as required. On average, patients in this study received 4 technique approaches during a single consultation; the minimum number of treatment approaches was 1 and the maximum number of treatment approaches was 11 (reported by only one practitioner). Advice given by the practitioner to the patient on diet, education, exercise, lifestyle and relaxation were considered to be education, or a similar activity, by the authors and were grouped under “education/advice”. The most commonly used treatment approaches were soft tissue (22.3%; n = 765), muscle energy technique (14.6%; n = 500), articulation (14.3%; n = 491) and education/advice (11.9%; n = 410) (Table 4).

**Table 4:** Treatment approaches for patients in this study

**Characteristic**	**Patient records n (%)**	**AOA census data n (%)**
**Suitability for treatment**		
Yes	795 (99.5)	n/a
No	4 (0.5)	n/a
**Treatment approaches used**		
Soft tissue^1^	765 (22.3)	1950 (22.7)
Articulation^2^	491 (14.3)	1278 (14.9)
HVLA Thrust^3^	340 (9.9)	1156 (13.4)
Cranial^4^	226 (6.6)	533 (6.20)
MET^5^	500 (14.6)	1123 (13.0)
Counterstrain^6^	275 (8.0)	395 (4.6)
Education/Advice	410 (11.9)	574 (6.7)
Exercise	196 (5.7)	741 (8.6)
Other*	226 (6.6)	856 (9.9)
No treatment	5 (0.1)	n/a

^1^A direct technique usually involving lateral or linear stretching, deep pressure, separation of muscle origin and insertion, and including myofascial release.

^2^A repetitive low velocity technique where a joint is carried through a range of motion with the aim to increase range of movement.

^3^High Velocity Low Amplitude technique, where a rapid therapeutic force (thrust) of brief duration over a short distance within a joint’s anatomical range of motion is employed to engage the restrictive barrier with the aim to decrease restriction in the joint.

^4^System of treatment using the primary respiratory mechanism (and balanced membranous tension).

^5^Muscle Energy Technique; form of treatment where the patient’s muscles are activated from a precisely controlled position, into a specific direction against a counterforce provided by the clinician.

^6^Strain-counterstrain; an indirect treatment method in which a myofascial tenderpoint is treated using passive positioning, resulting in spontaneous tissue release and decreased tenderness.

NOTE: all definitions were extracted from Glossary of Osteopathic Terms in *Foundations of Osteopathic Medicine*[13].

*Other treatment modalities include needling, stretching, ultrasound, visceral technique, functional technique, orthotics, balanced ligamentous tension, heat/ice application.

### Treatment outcomes

The number of treatments delivered to each patient was reported in 723 records, with the median number of treatments being 3 (min. 1, max. 20).

In this study, improvement was reported by 86.3% of those who returned after their first appointment (n = 746). By the end of their consultation period, 96.2% (n = 740) of patients had experienced improvement or resolution of the presenting complaint. Those patients reporting the treatment outcome as “best ever” or “resolved” increased from 7.2% (n = 54) after the first appointment, to 42.0% (n = 311) by the final appointment.

In this study, the overall number of complications following treatment, for example increased pain or stiffness, decreased from 19.2% (n = 142) after the first appointment, to 3.4% (n = 27) (Table 5). While there was no classification of severity, these complications are considered minor and usually would be expected with manual therapy [17]. No serious adverse events were reported.

**Table 5:** Treatment outcomes for patients in this study

**Outcome of treatment**	**First appointment n (%)**	**Final appointment n (%)**
Improved	338 (45.3)	121 (16.4)
Much improved	252 (33.8)	280 (37.8)
Best ever	21 (2.8)	100 (13.5)
Resolved	33 (4.4)	211 (28.5)
Worse	13 (1.7)	2 (0.3)
No change	89 (11.9)	26 (3.5)
**Complications of treatment**†	**First appointment n (%)**	**Subsequent appointments n (%)**
Increased pain	90 (12.2)	17 (2.3)
Increased stiffness	23 (3.1)	2 (0.3)
Dizziness	4 (0.5)	1 (0.1)
Nausea	2 (0.3)	1 (0.1)
Headache	10 (1.4)	2 (0.3)
Fatigue	13 (1.8)	4 (0.6)
None of these	598 (80.8)	700 (96.3)

**†**Reported complications occurred within 24–48 hours of first appointment, or those that continued to be reported after the second and subsequent appointments.

### Patient management

During the data collection period, approximately one in six (15.8%; n = 118) was referred to another practitioner, as part of the osteopath’s on-going management of the patient. Of those that provided information as to where the patient was referred (n = 86), the most common referral was to a general medical practitioner (GP) (33.7%; n = 29), a Pilates instructor (23.3%; n = 20) or a podiatrist (12.8%; n = 11). Other practitioners included another osteopath, acupuncturist, massage therapist, homeopath and counsellor.

The end result of the consultation period was reported in 96.4% of patient records (n = 770) (Table 6), with almost half of patients (48.4%; n = 373) recommended to return to the osteopath for episodic care; one quarter of patients (26.8%; n = 206) were discharged. A small number of patients were referred to another practitioner or for further investigations (4.6%; n = 35).

**Table 6:** Patient management

**Characteristic**	**Patient records (%)**
**End result of consultation period**	
Patient discharged	206 (26.8)
Maintenance treatment recommended	373 (48.4)
Referral for further investigations^	13 (1.7)
Referral to another practitioner	22 (2.9)
Still undergoing treatment	99 (12.9)
Terminated treatment- financial reasons	5 (0.6)
Terminated treatment – funding discontinued*	4 (0.5)
Did not return for treatment - unknown	48 (6.2)

^Patient still under the care of the osteopath; treatment pending results of requested investigations.

*Funding by insurance provider discontinued.

A number of patients in this study were still undergoing treatment for their presenting complaint at the end of the study period (12.4%; n = 99), possibly reflecting that data collection for some patients may have started late in the study period, therefore preventing completion of the patient data set within the time of the study. In 6.2% (n = 48), the patient did not return for treatment; the survey tool did not seek reasons for this outcome.

## Discussion

While osteopathy has been practiced in Australia since 1909 [5], is a government-regulated profession, and has experienced recent growth, it is still one of the smallest registered health professions in Australia. It is also relatively unknown, being used by less than 4% of the population [11]. It is likely that the low number of registered osteopaths contributes to the poor visibility of the profession in Australia (1,595 osteopaths compared to other registered professions: 22,384 physiotherapists; 4,350 chiropractors; 88,293 medical practitioners [16]).

This survey describes the current nature of osteopathic practice in Australia, in order to provide information to inform any discussions or debate on the scope of practice for Osteopathy in Australia.

### Participants

The number of osteopaths (n = 54) who contributed to this study represented 3.4% of nationally registered osteopaths in Australia at the time of data collection. While caution must be taken when interpreting the data in the present study, the demographic profile of osteopaths reported in this study was consistent with the findings of previous studies [8,9,12] and information published by the Australian Osteopathic Association and AHPRA regarding professional members [2,16].

As described in the existing literature [2,8,9,16], this study identified that the gender balance of participants is approximately equal, with a slightly more females. The majority of participants fell within the 30–39 year age range, and most had less than 15 years’ experience. Likewise, the state-wide distribution of osteopaths was similarly representative; the greatest concentration of registered osteopaths in Australia are in Victoria and NSW [16].

### Patient demographic information

The patients described by the participants were predominantly female (68%), consistent with the findings of previous research studies [9,10], and in common with health services utilisation trends overall [18]. Although the spread of ages for people attending osteopathic consultations was broad, the majority of patients were adults, with the median age of 39 years corresponding with the previous literature, whose median age range was found to be 30 to 49 years [9].

The proportion of referrals from general and registered practitioners was found by Orrock (2009) to be small but visible (GPs 4.9%, other practitioners 10.1%) [9]. The findings of this study reflected increasing referrals from GPs (7.3%) but does not approach the levels of referrals reported by Xue et al. (16%) [10], which may be a reflection of the small number of osteopathic patients (n = 51) who participated in that study.

The low proportion of referrals in this study supports the premise that osteopathy has a low visibility with other health professionals and indicates the need for increasing the awareness of the nature of osteopathic practice with other health professions. The main source of referrals for patients in this study was word-of-mouth, a similar finding in the previous literature [9,10].

### Clinical presentations

Consistent with previous studies, the prevailing presentations to osteopathic care in Australia in this study are musculoskeletal problems and are mainly spinal conditions [8-10]. The majority of people seeking osteopathic care in this study did so for an acute or sub-acute condition (63.6%), which varies from previously reported data that noted a high proportion of chronic presentations (51.2%) [9]. This shift in timing of seeing osteopaths for treatment in relationship to the occurrence of their complaint may reflect the increase in the availability of osteopathic care in line with the rapid growth of the profession in recent years.

This study also identified the range of co-morbid conditions that 37.9% of osteopathic patients presented with, and these details have not been reported in previous studies. The combination of depression and anxiety (10.5%) as mental health issues constitutes the second largest comorbidity of patients presenting to osteopaths in Australia, behind cardiovascular disorders. The diversity of coexisting complaints emphasises the need for osteopathic training to encompass the common medical considerations for these conditions, as well as developing the diagnostic skills to recognise the signs and symptoms of medical presentations requiring referral.

### Treatment approaches

While a wide scope of treatment approaches were reported to be used in this study, soft tissue techniques, MET and articulation techniques made up more than half of all treatment approaches. The primary choice of treatment modalities reflects the findings of the previous research [8,9]. Soft tissue technique, MET and articulation are fundamental osteopathic techniques taught very early in the training regime of novice osteopaths, and the dominant use of these techniques by the majority of practitioners may reflect the simplicity of the techniques, the ease with which they are implemented or possibly the efficacy of the techniques. These treatment approaches are generally considered to have a minimal risk associated with technique safety, and may account for the low number of adverse events and the high level of patient satisfaction reported. Education and advice was the fourth most common treatment approach used, reflecting the complex nature of osteopathic care.

### Treatment outcomes

Overall, patient outcomes to osteopathic care were positive in this study, with most records collected indicating overall improvement in the patient’s condition. The previous evidence suggested that patients seeking osteopathic care considered their treatment to be effective and a substantial number experienced reduction of pain (79.5%) [10].

The outcome of treatments reported by this study were very positive with 86.4% of patients reporting improvement after the first visit increasing to 96.2% reporting improvement by the final treatment, or at the end of the reporting period. For those patients whose treatments were completed to point of discharge, referred, or placed on maintenance schedule (75.2%), this result was achieved over a small number of treatments (median = 3); this result should be viewed conservatively, as it is practitioner-reported.

Maintenance treatment schedules are used for patients with underlying chronic conditions or circumstances such as occupational stresses that may lead to a repeat exacerbation of the presenting condition. The purpose of such treatments is to attempt to prevent reoccurrence or limit the severity of any reoccurrence of a particular complaint consistent with the preventative philosophy that is fundamental to osteopathic principles.

The frequency of complications to osteopathic treatment reported in this study was low, with the most significant reaction being increased pain after treatment. In acute presentations, it is not unusual to expect an exacerbation of symptoms after the initial treatment when using manual therapy techniques [17] such as soft tissue and articulation. The majority of patients did not experience any adverse reactions or events, and the number of complications was shown to decrease between the initial and final visits. This is likely to indicate both the low-risk nature of techniques commonly employed and the probability that the patient becomes accustomed to osteopathic treatment.

The treatment status question (SDC tool Item 54) was not answered in 25 cases of the 795 patients suitable for treatment, and 48 patients did not return for further treatment for reasons unknown; the most conservative interpretation is to assume that these patients (9.2%) were not satisfied with osteopathic care.

While these results should be viewed conservatively, as they are practitioner-reported, previous research has reported similar findings [10]. It must be noted that the severity of the complication was not asked and cannot be commented upon, and is a point for future research.

The considerable improvements reported after the initial and subsequent treatments would, within the limitations of this study, support the perception of clinical effectiveness of the osteopathic treatment approaches for the condition presenting in this study. The actual effectiveness of osteopathic clinical treatment would need to be established using standardised outcome measures assessed independently from the practitioner delivering the treatment.

Much of the research into Osteopathic Manipulative Treatment (OMT) has focused on proving efficacy of the manual treatment components of osteopathic practice by conducting randomised controlled trials (RCT) [19]. The diversity of treatment approaches, as indicated by range of modalities used in any particular clinical encounter would indicate the translation of strictly controlled RCTs to clinical practice would be very difficult. This study provides data to support, within limitations, the effectiveness and safety of clinical practice of OMT in Australia, and pragmatic trials of osteopathic practice should be undertaken to provide stronger evidence for osteopathic practice as a complex intervention to inform other health care practitioners and patients on the scope of osteopathy, its effectiveness and safety.

### Limitations

The data collection tool used in this study was useful in describing the activities of osteopaths in Australian practice. The tool was developed and trialled in the UK and was used as faithfully as possible to the original version. A number of omissions in the data were identified during analysis. Recording the ‘type’ of complaint and presentations other than pain could not be identified, and a specific diagnosis was not requested.

Another limitation of this study is that the practitioner, rather than the patient, reported the outcomes of treatment and there is potential bias in that the practitioner may be reporting on successful cases rather than all cases. Future studies should consider independently seeking patient feedback, and the viability of including audits of clinical records used in the data collection.

The method of this study was performed as set out in the guidelines given by NCOR [14]. The participation rate was low and extrapolating the results to the greater osteopathic population in Australia should be done with caution. Participants from all states and territories’ of Australia were not represented, with some states, such as Western Australia, Northern Territory and the ACT, not included, however the participants are statistically representative of distribution of osteopaths in Australia as the majority (77%) of registered osteopaths reside in Victoria and NSW [16].

Due to this low participation rate it is possible that the complete scope of osteopathic practice may not be covered; however the distribution of practitioners and other demographics are consistent with previously published studies [8,9,12].

## Conclusions

The nature of osteopathic practice in Australia as captured by the SCD tool has remained stable, despite recent rapid growth within the profession. In this study, osteopaths in Australia mainly see patients with acute or sub-acute musculoskeletal problems which are predominantly spinal conditions. A significant proportion of these patients have one or more significant coexisting condition, which was not their primary reason for seeking osteopathic treatment. The major coexisting conditions are cardiovascular, anxiety and depression, and respiratory symptoms. These conditions have not been previously reported in the literature on osteopathic practice in Australia.

The majority of patients experience a significant improvement within a small number of treatments (median 3) and complications were minor, with no major adverse events being reported.

There is a need for objective evidence to test these patient outcomes, and pragmatic trials of osteopathic practice should be undertaken to provide stronger evidence for the effectiveness and safety of osteopathic practice as a complex intervention.

## Abbreviations

AOA: Australian Osteopathic Association; AHPRA: Australian Health Practitioner Regulation Agency; MET: Muscle energy technique; NCOR: National Council for Osteopathic Research; OMT: Osteopathic manipulative treatment; RCT: Randomised controlled trial; SDC: Standardised data collection.

## Competing interests

The authors declare that they have no competing interests.

## Authors’ contributions

RM and SB were responsible for the conceptualisation of this project. AZ and RM were responsible for the data analysis. SB was the primary author of the manuscript, with substantial input from RM and AZ. SB and RM were responsible for altering the SDC tool for the Australian context and for the data collection and interpretation. AZ contributed to the data analysis and interpretation, critical review and revision of the manuscript. All authors approved the final version of the manuscript.

## Pre-publication history

The pre-publication history for this paper can be accessed here:

http://www.biomedcentral.com/1471-2474/14/227/prepub

## Supplementary Material

Additional file 1 NCOR Standard Data Collection Tool Australian Version. Click here for file
