# Supplementary material for: A profile of osteopathic practice in Australia 2010–2011: a cross sectional survey
Source: BMC Musculoskelet Disord. 2013 Aug 1;14:227. doi: 10.1186/1471-2474-14-227 (PMC3733950; doi:10.1186/1471-2474-14-227)
Supplement: Additional file 1 — NCOR Standard Data Collection Tool Australian Version. [file 1471-2474-14-227-S1.pdf]

NCOR

Standard Data Collection Tool

Australian Version

© The National Council for Osteopathic Research, 2009. Final version 2.1 Modified for Australia

Osteopath Name or code:..
(Optional, see Plain Language Statement)

Part 1: PATIENT FORM – Initial consultation details

To be completed by the osteopath

1. Date of first appointment

2. Gender: Male ☐ Female ☐

3. Postcode:

4. What is the patient's age?

5. What is the patient's height?

6. What is the patient's weight?

7. What is the patient's main occupation?

Not applicable ☐

8. How would you describe the patient's current work status? *(tick as appropriate)*

☐ Working full time (employed)
☐ Working full time (self-employed)
☐ Working part time (employed)
☐ Working part time (self-employed)
☐ Not currently employed
☐ Retired
☐ Student
☐ Pre-school

9. Does the patient receive disability allowance?

Yes ☐ No ☐ Not applicable ☐

10. Who referred the patient to this practice?*

☐ Patient
☐ GP
☐ Insurance company
☐ Employer
☐ Work cover
☐ Another healthcare practitioner
☐ Solicitor
☐ TAC
☐ EPC Plan

11. Has the patient ever had any osteopathic treatment before? ☐ yes ☐ no

12. How long did the patient have to wait for the <u>first appointment</u> to be offered?

☐ Same day
☐ 2-3 days
☐ 4-7 days
☐ 8 days or more

13. How many times has the patient visited their GP about this condition prior to coming to here?

times

14. How physically demanding is the patient's occupation?

☐ sedentary
☐ light
☐ moderate
☐ strenuous
☐ not applicable

15. How strenuous are the patient's leisure time activities? (see examples below)

☐ sedentary
☐ light
☐ moderate
☐ strenuous
☐ not applicable

EXAMPLE LEISURE ACTIVITIES
Sedentary: handicrafts, cinema
Light: badminton, bowling, light gardening, walking (including to and from shops)
Moderate: jogging, swimming, moderate gardening
Strenuous: basketball, competitive cycling, competitive swimming, football, squash, heavy gardening

© The National Council for Osteopathic Research, 2009. Final version 2.1 Modified for Australia

16. How many weeks has the patient had this current problem?

- ☐ less than 1 week
- ☐ 1-2 weeks
- ☐ 3-4 weeks
- ☐ 5-6 weeks
- ☐ 6-12 weeks
- ☐ 13 or more weeks

17. How many weeks has the patient been off work with this current problem?

- ☐ less than 1 week
- ☐ 1 week
- ☐ 2 weeks
- ☐ 3 weeks
- ☐ 4 weeks
- ☐ 5 weeks or more
- ☐ not applicable

18. How did the patient hear about this practice?*
(tick all that apply)

- ☐ Word of mouth/recommendation
- ☐ Local advert
- ☐ Yellow pages
- ☐ I live nearby
- ☐ From a healthcare practitioner
- ☐ Internet search
- ☐ Other, please specify

19. Why did the patient decide to have osteopathy?* (tick all that apply)*

- ☐ Personal recommendation or referral
- ☐ Personal research
- ☐ Failure of previous treatment
- ☐ Previous experience of osteopathic treatment
- ☐ Desire to have osteopathic treatment
- ☐ Wanted a form of manual or hands on treatment
- ☐ Wanted to have drug-free treatment
- ☐ Other, please specify

20. Is the patient on a Hospital waiting list for treatment for this condition?*

☐ yes ☐ no

21. How long has the patient been waiting for Hospital treatment for this condition?*

_____Weeks Not applicable ☐

22. Has the patient had previous Medical/Hospital treatment for this episode of this condition?*

Yes ☐ **No** ☐

If yes, has this included: *Tick all that apply*

- ☐ Imaging e.g. an X-Ray or scan
- ☐ Medication
- ☐ Hospital outpatient treatment
- ☐ Hospital inpatient treatment

23. How would you classify the ethnic background of this patient?

Oceanian
- ☐ Australian
- ☐ Australian Aboriginal
- ☐ Torres Straight Islander
- ☐ Australian South Sea Islander
- ☐ New Zealander
- ☐ Maori
- ☐ Other……………………………………..

North West European
- ☐ English
- ☐ Scottish
- ☐ Irish
- ☐ Dutch
- ☐ German
- ☐ Other ………………………………………

Southern and Eastern European
- ☐ Italian
- ☐ Spanish
- ☐ Greek
- ☐ Croatian
- ☐ Other……………………………………..

North African and Middle Eastern
- ☐ Arab
- ☐ Jewish
- ☐ Other……………………………………..

South-East Asian
- ☐ Vietnamese
- ☐ Indonesian
- ☐ Other…………………………………………

North-East Asian
- ☐ Chinese
- ☐ Other…………………………………………

Southern and Central Asian
- ☐ Indian
- ☐ Pakistani
- ☐ Other…………………………………………

People of the Americas
- ☐ North American
- ☐ Central American
- ☐ South American
- ☐ Other…………………………………………

Sub-Saharan African
- ☐ Central and West African
- ☐ Southern and East African

- ☐ Other…………………………………………

© The National Council for Osteopathic Research, 2009. Final version 2.1 Modified for Australia

24. How long has the patient had their current symptoms with this episode?

25. Type of onset of symptoms? *Tick all that apply*

- ☐ Acute/sudden onset (of unknown origin)
- ☐ Traumatic onset (of known origin)
- ☐ Slow/insidious onset
- ☐ Recurring problem

26. Symptom areas: Please record up to three predominant symptom areas in order of priority

1. ☐ **2.** ☐ **3.** ☐

1	Head/facial area	12	Lumbar
2	Temporo-mandibular	13	Sacroiliac/pelvis/groin
3	Neck	14	Gluteal region
4	Shoulder	15	Hip
5	Upper arm	16	Thigh/upper leg
6	Elbow	17	Knee
7	Forearm	18	Lower leg
8	Wrist	19	Ankle
9	Hand	20	Foot
10	Thoracic spine	21	Abdomen
11	Rib cage	22	Other ………………

27. Is this the first episode? *Please tick*

- ☐ Yes, first time onset
- ☐ Second episode
- ☐ Third episode
- ☐ Fourth or more episodes

28. What investigations have taken place for this current problem? *Tick all that apply*

- ☐ None
- ☐ Blood test
- ☐ X-Ray
- ☐ CT Scan
- ☐ MRI
- ☐ Ultrasound scan
- ☐ Urinalysis
- ☐ Other (please state)

29. What current co-existing conditions (diagnosed by a medical practitioner) does the patient have (tick all that apply)

- ☐ Anaemia
- ☐ Angina
- ☐ Anxiety
- ☐ Arthritis
- ☐ Asthma
- ☐ Bowel disease
- ☐ Cancer
- ☐ COPD (chronic obstructive pulmonary disease)
- ☐ CHF (Congestive heart failure)
- ☐ Dementia
- ☐ Depression
- ☐ Diabetes
- ☐ Hearing impairment
- ☐ Hypertension
- ☐ Kidney disease
- ☐ Liver disease
- ☐ Migraine
- ☐ MI (myocardial infarct)
- ☐ Neurological disease
- ☐ Osteoporosis
- ☐ Peripheral vascular disease
- ☐ Pregnancy
- ☐ Stroke/TIA (Transient Ischaemic Attack)
- ☐ Upper gastrointestinal disease
- ☐ Visual impairment
- ☐ Other (please state)

© The National Council for Osteopathic Research, 2009. Final version 2.1 Modified for Australia

Part 2: Management and treatment

First appointment

30. Was this patient suitable for osteopathic treatment? Yes ☐ No ☐

31. What types of treatment approaches have been used with the patient today?

- ☐ No treatment
- ☐ Soft tissue
- ☐ Articulation
- ☐ HVLA thrust
- ☐ Cranial techniques
- ☐ Muscle energy
- ☐ Strain/counterstrain
- ☐ Functional technique
- ☐ Visceral
- ☐ Myofascial release (MFR)
- ☐ Education
- ☐ Relaxation advice
- ☐ Steroid Injection
- ☐ Acupuncture
- ☐ Dietary advice
- ☐ Exercise
- ☐ Orthotics
- ☐ Lifestyle advice
- ☐ Other (please name)

32. Was informed consent obtained for any particular technique used?

Yes ☐
Technique (*Please state)*

…………………………………

No ☐
Not applicable ☐

33. How was consent gained from the patient about osteopathic examination and treatment? (*Tick all that apply)*

- ☐ Implied consent
- ☐ Verbally
- ☐ Written
- ☐ Written and verbal
- ☐ Not applicable
- ☐ Other

34. Has information concerning the following been discussed with the patient?

Possible risks of examination and treatment	Yes ☐ No ☐
Possible side-effects of treatment	Yes ☐ No ☐

35. What other education and advice have been given to the patient to inform them about their condition? *Please record all that apply*

- ☐ Explanation of presenting problem
- ☐ Possible risk factors associated with a recurrence of symptoms
- ☐ Anticipated response to treatment
- ☐ Anticipated number of treatments
- ☐ Not applicable

36. What self-management strategies have been recommended for the patient to use?* *Please tick all that apply*

- ☐ None
- ☐ Application of heat
- ☐ Application of cold
- ☐ Stretches
- ☐ Rest
- ☐ Specific exercise
- ☐ General exercise
- ☐ Other (please state)
- ☐ Vitamin or other nutritional supplements
- ☐ Postural exercises/advice
- ☐ Natural remedies
- ☐ Relaxation advice
- ☐ Advice concerning physical activity
- ☐ Homeopathic or Naturopathic remedies
- ☐ Other

……………………………………………

© The National Council for Osteopathic Research, 2009. Final version 2.1 Modified for Australia

37. Who is responsible for payment for treatment *

- ☐ Self
- ☐ Insurance company
- ☐ Employer/own company
- ☐ Other (please state)

38. Is an insurance case or litigation claim pending?

Yes ☐ No ☐

39. Time allocated for first appointment __________ **minutes**

Second and subsequent appointments

40. After the <u>first</u> appointment, did the patient report any of the known complications of treatment described below within the first 24-48 hours?

- ☐ None of these
- ☐ Increased pain
- ☐ Increased stiffness
- ☐ Dizziness
- ☐ Nausea
- ☐ Headache
- ☐ Fatigue
- ☐ Serious adverse event, if known, please describe below

41. What was the patient's overall outcome after the <u>first</u> appointment?*

- ☐ Worst ever
- ☐ Much worse
- ☐ Worse
- ☐ Not improved/not worse
- ☐ Improved
- ☐ Much improved
- ☐ Best ever
- ☐ Resolved

42. What types of treatment approaches have been used with the patient? *Please tick all that apply*

- ☐ No treatment
- ☐ Soft tissue
- ☐ Articulation
- ☐ HVLA thrust
- ☐ Cranial
- ☐ Muscle energy
- ☐ Strain/counterstrain
- ☐ Functional
- ☐ Visceral
- ☐ Myofascial release (MFR)
- ☐ Education
- ☐ Relaxation advice
- ☐ Steroid Injection
- ☐ Acupuncture
- ☐ Dietary advice
- ☐ Exercise
- ☐ Orthotics
- ☐ Other (please name)

43. Was informed consent obtained for any particular technique used?

Yes ☐

Technique (*Please state)*

……………………………………

No ☐

Not applicable ☐

44. How was consent gained from the patient about osteopathic examination and treatment? (*Tick all that apply)*

- ☐ Implied consent
- ☐ Verbally
- ☐ Written
- ☐ Written and verbal
- ☐ Not applicable
- ☐ Other

© The National Council for Osteopathic Research, 2009. Final version 2.1 Modified for Australia

45. What other education and advice have been given to the patient to inform them about their condition? *Please record all that apply*

- ☐ Explanation of presenting problem
- ☐ Possible risk factors associated with a recurrence of symptoms
- ☐ Anticipated response to treatment
- ☐ Anticipated number of treatments
- ☐ Not applicable

46. What self-management strategies have been recommended for the patient to use? *Please tick all that apply**

- ☐ None
- ☐ Application of heat
- ☐ Application of cold
- ☐ Stretches
- ☐ Rest
- ☐ Specific exercise
- ☐ General exercise
- ☐ Other (please state)
- ☐ Vitamin or other nutritional supplements
- ☐ Postural exercises/advice
- ☐ Natural remedies
- ☐ Relaxation advice
- ☐ Advice concerning physical activity
- ☐ Homeopathic or Naturopathic remedies
- ☐

47. Time allocated for follow up appointments **minutes**

Part 3: Final outcome report

48. Date of final visit:

49. Did the patient continue to report any of the known complications of treatment described below after the second and subsequent appointments?

- ☐ None of these
- ☐ Increased pain
- ☐ Increased stiffness
- ☐ Dizziness
- ☐ Nausea
- ☐ Headache
- ☐ Fatigue
- ☐ Serious adverse event, if known, please describe

50. What was the patient's overall outcome at their final appointment or to date?

- ☐ Worst ever
- ☐ Much worse
- ☐ Worse
- ☐ Not improved/not worse
- ☐ Improved
- ☐ Much improved
- ☐ Best ever
- ☐ Resolved

51. How many treatments did it take to achieve this?

© The National Council for Osteopathic Research, 2009. Final version 2.1 Modified for Australia

52. Did you contact the patient's GP during this course of treatment?	53. How many treatments did the patient have before being able to return to work (if applicable)?
☐ Yes since patient was referred to practice by GP<br>☐ Yes since GP had requested information<br>☐ Yes to request further information or investigation<br>☐ Yes for referral for other treatment<br>☐ No, the patient's GP was not contacted	[ ] Treatments<br><br>☐ Not applicable

54. What was the end result of the consultation period?*

- ☐ No further treatment. The patient was discharged.
- ☐ Patient was recommended to return for episodic care (Maintenance).
- ☐ Patient was referred for further investigations pending treatment under the care of the practice.
- ☐ Patient was referred on
- ☐ The patient terminated treatment due to lack of further personal funds
- ☐ The patient terminated treatment due to lack of further funding by insurance providers
- ☐ The patient did not return – reason unknown
- ☐ The patient is still undergoing treatment for this condition at the end of the study period

55. Was the patient referred on from the practice? Yes ☐ No ☐
If yes, where was the patient referred to? Tick one option.

- ☐ Their GP for further investigations
- ☐ Their GP to try and arrange other treatment
- ☐ Another osteopath
- ☐ A homeopath
- ☐ An acupuncturist
- ☐ A podiatrist
- ☐ An Alexander teacher
- ☐ A physiotherapist
- ☐ A counsellor
- ☐ A chiropractor
- ☐ A sports massage therapist
- ☐ A Pilates trainer
- ☐ Other (please state)

Thank you for completing this form

Statement of accreditation

"This standardised data collection tool has been produced by the National Council for Osteopathic Research (NCOR), and funded by the General Osteopathic Council (GOsC), the UK regulator of osteopaths. The intellectual property rights in the standardised data collection tool are jointly owned by the NCOR and the GOsC. The tool should be referenced in published work as: Moore AP, Leach CMJ, Fawkes CA. Standardised data collection tool for osteopathic practice. National Council for Osteopathic Research (UK) and General Osteopathic Council UK, 2009".

*This tool has been modified with permission for use within Australia (Discipline of Osteopathy, RMIT University, Bundoora, April 2010)

© The National Council for Osteopathic Research, 2009. Final version 2.1 Modified for Australia
